# Safety of Autologous Cord Blood Cells for Preterms: A Descriptive Study

**DOI:** 10.1155/2018/5268057

**Published:** 2018-08-15

**Authors:** Jie Yang, Zhuxiao Ren, Chunyi Zhang, Yunbei Rao, Junjuan Zhong, Zhu Wang, Zhipeng Liu, Wei Wei, Lijuang Lu, Jiying Wen, Guocheng Liu, Kaiyan Liu, Qi Wang

**Affiliations:** ^1^Department of Neonatology, Guangdong Women and Children Hospital, Guangzhou, China; ^2^Guangdong Cord Blood and Stem Cell Bank, Guangzhou, China; ^3^Department of Obstetrics, Guangdong Women and Children Hospital, Guangzhou, China; ^4^Institute of Hematology, People's Hospital, Peking University, Beijing, China

## Abstract

**Background:**

Preterm birth complications are one of the leading causes of death among children under 5 years of age. Despite advances in medical care, many survivors face a lifetime of disability, including mental and physical retardation, and chronic lung disease. More recently, both allogenic and autogenic cord blood cells have been applied in the treatment of neonatal conditions such as hypoxic-ischemic encephalopathy (HIE) and bronchopulmonary dysplasia (BPD).

**Objective:**

To assess the safety of autologous, volume- and red blood cell- (RBC-) reduced, noncryopreserved umbilical cord blood (UCB) cell infusion to preterm infants.

**Method:**

This study was a phase I, open-label, single-arm, single-center trial to evaluate the safety of autologous, volume- and RBC-reduced, noncryopreserved UCB cell (5 × 10^7^cells/kg) infusion for preterm infants <37 weeks gestational age. UCB cell characteristics, pre- and postinfusion vital signs, and laboratory investigations were recorded. Clinical data including mortality rates and preterm complications were recorded.

**Results:**

After processing, (22.67 ± 4.05) ml UCB cells in volume, (2.67 ± 2.00) × 10^8^ cells in number, with (22.67 ± 4.05) × 10^6^ CD34+, (3.72 ± 3.25) × 10^5^ colony forming cells (CFU-GM), and (99.7 ± 0.17%) vitality were infused to 15 preterm infants within 8 hours after birth. No adverse effects were noticed during treatment. All fifteen patients who received UCB infusion survived. The duration of hospitalization ranged from 4 to 65 (30 ± 23.6) days. Regarding preterm complications, no BPD, necrotizing enterocolitis (NEC), retinopathy of prematurity (ROP) was observed. There were 1/15 (7%) infant with intraventricular hemorrhage (IVH), 5/15 (33.3%) infants with ventilation-associated pneumonia, and 10/15 (66.67%) with anemia, respectively.

**Conclusions:**

Collection, preparation, and infusion of fresh autologous UCB cells to preterm infants is feasible and safe. Adequately powered randomized controlled studies are needed.

## 1. Introduction

Preterm delivery is a global health problem. The rate of preterm birth ranges from 5% to 18% of babies born across 184 countries. An estimated 15 million babies are born preterm every year [1]. Preterm birth complications are the leading cause of death among children under 5 years of age, which are responsible for nearly 1 million deaths in 2015. The morbidity associated with preterm birth often extends to later life, resulting in enormous physical, psychological, and economic costs [2]. Inflammation, ischemia, and free radical toxicity lead to multiorgan damage in preterm infants, characterized by reduced numbers of tissue cells, blood vessels, and progenitor cell [3–6]. Current management has been shown to reduce preterm complications and overall morbidity. However, many survivors still face a lifetime of disability, including mental and physical retardation, and chronic lung disease [1]. It has been reported that among infants born with gestational ages of 22 to 28 weeks, 16% are complicated with severe intraventricular hemorrhage (IVH), 36% with late-onset sepsis (LOS), and 68% with bronchopulmonary dysplasia (BPD) [7]. The current treatments such as pulmonary surfactant administration, noninvasive respiratory support, and antibiotic administration are single-organ or symptom-targeted. Neonatologists are in urgent need for new systemic multiorgan-targeted treatments.

Human umbilical cord blood cells (UCBC) are abundant in stem cells. These primitive cells can home into damaged tissues, produce anti-inflammatory and immune-modulatory factors by paracrine effects, and differentiate into tissue cells [8]. Potential effects on respiratory distress syndrome (RDS), sepsis, and hypoxic-ischemic brain damage have been suggested in animal models [9–11]. Recently, these potential effects have been proved to be safe and feasible in clinical applications. Allogenic umbilical cord blood-derived mesenchymal stem cells (MSCs) have been applied in adults with acute RDS [12] and preterm infants with BPD [13], and autologous UCBC has been applied to neonates with HIE [14].

Recently, delayed cord clamping in premature neonates have been reported to improve neonatal mortality and morbidity. The American College of Obstetricians and Gynecologists now recommends a delay in umbilical cord clamping in preterm infants for at least 30–60 seconds after birth [15]. The potential mechanism was that delayed cord clamping was accompanied by an increased supply of RBCs and valuable progenitor cells [16].

Based on these evidence, we hypothesized that autologous cord blood infusion was safe for preterm infants. We report the outcomes of the infusion of autologous, volume- and RBC-reduced, noncryopreserved cord blood cell to 15 premature neonates.

## 2. Methods

This study was a phase I, open-label, single-arm, single-center trial to evaluate the safety of autologous, volume- and red blood cell- (RBC-) reduced, noncryopreserved umbilical cord blood cells (UCBC) (5 × 10^7^cells/kg) infusion for preterm infants <37 weeks gestational age.

### 2.1. Patients

We initiated this pilot study in December 2009. Inborn infants admitted to the Neonatal Intensive Care Unit (NICU) of Guangdong Women and Children's Hospital were eligible if they were (1) preterm: <37 weeks gestation, (2) without congenital abnormalities, (3) without maternal chorioamnionitis, (4) had available UCB, and (5) the mother was negative for hepatitis B (HBsAg and/or HBeAg) and C virus (anti-HCV), syphilis, HIV (anti-HIV-1 and -2) and IgM against Cytomegalovirus, rubella, toxoplasma, and herpes simplex virus. The study protocol was approved by the ethics committee of Guangdong Women and Children's Hospital. All patients in the study were given an intensive care therapy in accordance with the departmental guidelines which included therapies including positive pressure mechanical ventilation, noninvasive respiratory support, oxygen therapy, and exogenous surfactant (Curosurf, Chiesi, Parma, Italy) replacement. Chest radiographs were performed at admission and 8 hours after CBT on the first day of life in all surviving patients. Blood gas was monitored every 24 hours until weaning from ventilation. All clinical diagnoses were defined according to a standard reference [17]. Soon after the preterm infant was delivered, written consent was signed by the parents, and autologous cord blood infusion was applied to the baby in addition to routine pulmonary surfactant replacement and mechanical ventilation support as indicated.

### 2.2. Cord Blood Process

Guangdong Cord Blood and Stem Cell Bank is a public provincial blood bank affiliated to the Guangdong Women and Children's Hospital, which collects cord blood of every delivery in this hospital. Therefore, the cord blood of all the subjects had been routinely collected during the delivery. The procedure of cord blood collection and transfusion was performed in accordance with the cord blood bank guidance [18]. The umbilical cord was clamped for the collection using a blood-collection bag (WEGO, China) containing 28 ml of citrate-phosphate-dextrose anticoagulant right after the baby was born and before the placenta was delivered. The umbilical vein was sterilized and punctured with a 17-gauge needle. UCB collections were made by trained obstetricians or cord blood bank collection staff who were present at the hospital during weekdays for 8–12 hours per day. When collection was completed, the blood bag tubing was closed and sealed. Cord blood labeled with the full name of the donor, group type, and volume of the blood product was stored in 4 degree and sent to the Cord Blood and Stem Cell Bank for processing immediately. Before processing, 2 ml samples were taken from all collected CB units to test for the presence of virus (human immunodeficiency virus, hepatitis B virus, hepatitis C virus, and Cytomegalovirus) and bacterial infections (including Treponema Pallidum). A sample of peripheral blood was collected from the mother and tested for the presence of maternal transmissible diseases. And the results were obtained soon before the transfusion started. After the sample was taken, it was volume- and RBC-reduced after 30 minute incubation with 6% Hespan (Bethlehem, USA) following established CBB procedures using the SEPAX S-100 automated processing system (Biosafe, Geneva, Switzerland) if the unit contained >30 ml of UCB or manually if the unit was <30 ml. The mononuclear layer was isolated by density gradient centrifugation (1000*g*, 30 min, RT, Beckman, American), then was transferred to cryobags. Excessive nucleated cell-poor plasma was expelled. Meanwhile, MNC count, CD34 cell, CFU-GM, and sterility detection (Sheldon Manufacturing Inc., Cornelius, OR, USA) were performed. Cell viability was measured via 7-aminoactinomycin D (7-AAD) detection kit through flow cytometry analysis (BD Bioscience, USA). All infusions were administered in Guangdong Women and Children Hospital. Infusate and subject identities were double-checked by the research and clinical nursing staff. Infusions were also monitored by the research and clinical staff. Cells were infused over 15 minutes, followed by a 2 ml saline flush to clear the intravascular line.

#### 2.2.1. Assessment of Safety

Shortly, before, during, and until 24 hours after transfusion, heart rate, systolic, diastolic, and mean arterial blood pressure and arterial blood oxygen saturation level were monitored in peripheral blood continually and documented. Moreover, laboratory investigations in peripheral blood were monitored and kept stable during the whole treatment period, detailed in Table 1. Infusion reactions and signs of circulatory overload were checked.

#### 2.2.2. Results

From January 1, 2009, till June 5, 2016, fifteen infants were enrolled for the treatment, gestational age ranged from 28 2/7 to 34 1/7 (31.2 ± 1.62) weeks and birth weight ranged from 1200 to 2220 (1582.7 ± 252.8) grams; 12/15 (80%) were delivered by cesarean section. All 15 patients who received the cord blood infusion survived. The duration of hospitalization ranged from 4 to 65 (30 ± 23.6) days. Details were shown in Table 2.

### 2.3. Characteristics of Cord Blood Processing

Cord blood volume collected ranged from 27 to 76 ml, mean (47.13 ± 19.10) ml; volume postprocessing ranged from 16 to 30 ml, mean (22.67 ± 4.05) ml; cells collected ranged from 0.97 to 8.11 (×10^8^), mean (3.10 ± 2.17 × 10^8^); cells postprocessing ranged from 0.86 to 7.83 (×10^8^), mean (2.67 ± 2.00 × 10^8^); cells concentration postprocessing ranged from 5.85 to 40.8 × 10^6^/ml, mean (13.10 ± 10.35 × 10^6^/ml); CFU-GM ranged from 0.72 to 11.27 (×10^5^), mean (3.72 ± 3.25 × 10^5^); amount of CD34+ cells in units varied widely, ranged from 0.1 to 16.22 × 10^6^, mean (22.67 ± 4.05) ml; and viability of postprocessing units was high, ranged from 99.5 to 100%, mean (99.7 ± 0.17%). Details are shown in Table 3.

### 2.4. Infusion

Infused NC ranged from 4.48 to 5.0 × 10^7^/kg, mean (4.97 ± 0.13 × 10^6^/ml); time between collection (birth) and initiation of infusion ranged from 4.5 to 9 hours after birth, mean (6.77 ± 1.52 h); infused volume ranged from 2 to 28 ml, mean (10.27 ± 6.18 ml); pathogen detection (including bacteria culture, fungus culture, human immunodeficiency virus, hepatitis B virus, hepatitis C virus, Cytomegalovirus, and Treponema Pallidum) results were all negative.

### 2.5. Cord Blood Safety

The patient's vital signs and laboratory investigations were monitored during the whole treatment period, details were shown in Table 1. No significant infusion reactions were noted. No signs of circulatory overload and graft-versus-host disease (GVHD) were detected. Heart rate, mean arterial pressure, and oxygen saturation did not vary significantly before and after infusions.

### 2.6. Clinical Presentation and Complications

#### 2.6.1. Mortality

The fifteen patients who received infusions all survived.

#### 2.6.2. Nervous System

Three patients had birth asphyxia, among them one suffered from IVH. None of the patients developed abnormal clinical features of central nervous system disorders such as convulsions, apnea, or dysphagia.

#### 2.6.3. Respiratory System

12/15 (80%) presented with tachypnea and grunting soon after birth. The infants were diagnosed with RDS, 2/15 (13.3%) cases were grade I, 5/15 (33.3%) cases were grade II, 6/15 (40%) cases were grade III, and 2/15 (13.3%) cases were grade IV; and 12/15 (80%) received one dose PS replacement and 8/15 (53.3%) received intubation-surfactant replacement extubation–nasal continuous positive airway pressure (INSURE) therapy; however, one patient needed reintubation. 4/15 (26.7%) received mechanical ventilation; the median duration was 3.2 ± 1.8 days. The duration of oxygen therapy was (5.3 ± 3.0) days. No patient suffered from BPD, and chest radiographs showed improvement.

#### 2.6.4. Infection

5/15 suffered from ventilation-associated pneumonia (VAP), of which two were cases of Klebsiella pneumonia, one was a case of *Pseudomonas aeruginosa* pneumonia, one was a case of *Acinetobacter baumannii* pneumonia, and 1 suffered from late onset sepsis, infected with Klebsiella pneumonia proved by blood culture.

#### 2.6.5. ROP

No patients suffered from ROP.

#### 2.6.6. NEC

No patients suffered from NEC.

#### 2.6.7. Anemia

10/15 (66.67%) suffered from anemia (≤140 g/l); 2/15 (13.33%) needed RBC transfusion.

## 3. Discussion

In our study, we treated 15 preterm infants with autologous, volume and RBC-reduced cord blood cells. The treatment was started within 8 hours after birth. No adverse effect of cell therapy was noticed. No patient died during treatment. No preterm complications such as BPD, NEC, or ROP were observed. Our study presents preliminary data on the safety of autologous cord blood cell therapy in preterm infants. We postulated that several factors contributed to the safety issue, among them, the most important one was the autologous cell source. Based on the autologous cell source, no GVHD-related complication was observed. Moreover, autologous cell source avoided ethical issues. A second factor that contributed to the safety issue was cord blood minimal-processing procedure. In our study, only density gradient centrifugation was employed to separate nucleated cells. Since our cell infusions were started within 8 hours after birth, no cryopreservation was needed; thus, no chemicals were added into the cord blood cells for cryopreservation. This minimal-processing procedure and immediate transfusion after processing helped to avoid contamination and possible chemical toxicity. It also alleviated decrease of viability which may happen during storage.

In our study, mean (47.13 ± 19.10) ml cord blood with a total TNC of (3.10 ± 2.17) × 10^8^ mononuclear cells was collected before processing. After processing, cord blood volume and TNC were reduced to (22.67 ± 4.05) ml and (2.67 ± 2.00) × 10^8^, respectively, including (22.67 ± 4.05) × 10^6^ CD34+ and (3.72 ± 3.25) × 10^5^colony forming cells (CFU-GM) in a vitality (99.7 ± 0.17%).

Recently, delayed umbilical cord clamping 30–60 seconds after birth in preterms had been recommended by the American College of Obstetricians and Gynecologists and had been reported to reduce preterm-related complications. It is possible that delayed cord clamping increases supply of RBC and valuable stem and progenitor cells (SPC), thus may improve mortality and morbidity in premature neonates [15, 16]. However, delayed umbilical cord clamping mainly supply RBC instead of MNC to the infants. Therefore, we used noncryopreserved autologous cord blood cells infusion soon after preterm birth which contains mainly MNC with a lower volume. As is known that SPC mainly exits in MNC layer. It is considered as the most effective component in cord blood and strongly associated with a lower risk of developing preterm complications [19, 20]. Further, in view of the vulnerable heart function of preterm infants, they could only accept limited volume of infusion. Therefore, to achieve more MNC in a lower volume, we used volume- and RBC-reduced cord blood cells in our study to decrease the burden to the heart.

BPD is the main complication that contributes to morbidity and mortality in extremely premature newborns. It has been reported that among infants born with gestational ages of 22 to 28 weeks, 68% suffered from BPD [7]. The pathophysiologic features of BPD include abnormal lung growth characterized by reduced numbers of alveoli, blood vessels, prominent fibrosis, and secondary pulmonary hypertension [21]. Cord blood angiogenic progenitor cells and endothelial progenitor cells were reported to be decreased in preterm infants with BPD [5, 6]. Current therapy for BPD included noninvasive ventilation strategies, inhaled nitric oxide, antioxidants, vitamin A, caffeine, and corticosteroids. However, so far there are no specific therapies that have been widely adopted. It has been reported that MSCs may release anti-inflammatory paracrine factors. These factors have effect on both lung injury and sepsis [22, 23]. Periventricular leukomalacia (PVL) is another severe preterm complication, affecting 16% infants born with gestational age of 22 to 28 weeks [7]. PVL reflected perinatal damage from inflammation and oxidation to the developing brain, which was one of main reasons responsible for cerebral palsy [24]. Current therapy for hypoxic-ischemic damage was hypothermia. However, hypothermia therapy is contraindicated in preterm infants because of their immature thermoregulation [25, 26]. HUCBC has been shown to be effective in newborn models of hypoxic injury [4]; furthermore, autologous intravenous UCB infusion is safe and feasible in neonates with HIE and young children with acquired neurological disorders [7, 27]. In our study, there was one case complicated with IVH; however, no clinical presentation related to the central nervous system was observed. The underlying mechanism might be that the UCBC migrate to damaged sites, form anti-inflammatory or immunomodulatory factors, then proliferate into neurons [28, 29]. Sepsis is a common and major cause of death in preterm infants [30, 31]. Among very low birth weight infants (VLBW; < 1500 g), rates of sepsis range between 11% and 46% [32]. Neonatal sepsis and systemic inflammatory response syndrome (SIRS) are associated with brain damage [31, 33]. Current therapy for neonatal sepsis is the antibiotic administration. However, antibiotic resistance is a therapeutic problem in preterms. Substantial evidence from models of both lung injury and sepsis suggested that MSCs have an anti-inflammatory effect on host tissue, partly through the release of paracrine factors, reprogramming host macrophages [22, 23, 34]. In addition, MSCs reduced alveolar bacterial counts and improved alveolar macrophage phagocytosis after direct bacterial injury mediated by FGF7, LL-37 [35, 36]. All these investigations have laid down the foundation for cord blood cell therapy for preterms with pulmonary disorders complicated with sepsis and brain damage. NEC and ROP are two main complications of preterm infants; however, there were no cases observed in our study; the underlying reason might be the limited enrolled number and relatively large gestational age of infants enrolled in our study. To achieve more evidence regarding NEC and ROP, a large cohort study will be needed in our future study.

Studies on safety and feasibility of whole autologous cord blood transplant in preterm were also reported [19, 20]. Rudnicki and colleagues compared whole autologous cord blood infusion with allogeneic red blood cells in the treatment of preterm with anemia and showed autologous CB infusion was as effective and safe as allogeneic RBC transfusion [19]. In our study, 10/15 (66.67%) infants suffered from anemia (≤140 g/l); 2/15 (13.33%) needed RBC transfusion. Allogeneic RBCs transfusion is the main therapy for severe anemia [17]. In this study, we explore the safety of volume- and RBC-reduced cord blood cells to treat preterm infants. Both autologous cord blood infusion possess its advantage. However, further multiple center randomized controlled studies are needed regarding short-term and long-term outcomes.

Regarding the administration route, there were reports on the advantage of damaged site administration when compared to intravenous infusion. However, on the one hand, the potential preterm complications were due to multiorgan damage. To achieve the multiorgan-targeted effect, we used intravenous infusion as the administration route, which may result in cells being trapped in organs such as lung and brain. On the other hand, in the report supported site administration, allogenic-MSC was used in intratracheal administration to treat hyperoxia-induced lung damage; it seemed to attenuate the side effects of rejection.

In our study, we chose the infusion timing to be very soon after birth which is within the first 8 postnatal hours. Although some infants were delivered at midnight, we tried to process as early as within 8 hours after their birth. As it had been reported that it would take more than 1 week for progenitor cells to differentiate into damaged tissue cells, we administered CBC during the first hours after birth, so that it might provide enough time for these cells for differentiation.

In conclusion, we demonstrated autologous, volume- and RBC-reduced, noncryopreserved cord blood cells transfusion soon after birth was safe and feasible in preterm infants. Autologous cord blood infusion avoid GVHD; meanwhile, the reduced volume would protect the fragile cardio-function of the preterm infants. This autologous, volume- and RBC-reduced, method guaranteed the safety of application. In addition, this was an autologous transfusion instead of “cell transplantation therapy”, and therefore it was not regulated by the FDA regulation licensing public cord blood banks distributing unrelated banked cord blood units for allogeneic transplantation in 2012. However, our study was the single-center descriptive study with limited number of preterm infants, further multicenter randomized controlled trials are needed to prove the effectiveness.

## Figures and Tables

**Table 1:** Clinical findings previous and post infusion.

Serial number	Blood routine								Blood gas											
	HB		HCT		WBC		PLT		pH			PO2			PCO2			Fio2		
	Pre	Post	Pre	Post	Pre	Post	Pre	Post	Pre	Post-12 h	Post-24 h	Pre	Post-12 h	Post-24 h	Pre	Post-12 h	Post-24 h	Pre	Post-12 h	Post-24 h
1	177	144	50	37.3	10.8	9.2	304	335	7.34	7.27	7.45	9.3	8.9	8.9	4.8	7	3.2	0.3	0.21	0.21
2	167	137	49	37.4	10.9	10.2	284	243	7.26	7.4	7.37	6.7	12.7	6.4	6.8	3.7	4.6	0.4	0.25	0.21
3	135	132	37	15.3	14.9	13.2	252	248	7.25	7.2	7.33	4.64	5.07	9.9	7.25	8.02	4.26	0.35	0.25	0.21
4	145	144	39	39.9	8.8	10.1	405	336	7.25	7.28	7.39	6.5	9.1	6.1	7.6	6.6	4.4	0.35	0.21	0.21
5	171	180	50	50.6	9.6	17.9	265	161	7.42	7.39	7.34	3.9	4.5	6.4	3.7	3.9	5.1	0.55	0.3	0.23
6	120	116	35	34	14	13.5	320	256	7.34	7.36	7.49	8.3	6.6	5.6	5.9	4.8	3.2	0.45	0.21	0.21
7	148	139	44	39.1	35	14.2	274	446	7.3	7.34	7.37	11.6	11.3	6.95	5.1	4.28	4.34	0.35	0.21	0.21
8	212	203	60.5	54.3	13.7	14	413	420	7.42	7.28	7.45	10.48	10.25	8.31	4.78	4.82	3.62	0.25	0.21	0.21
9	149	136	43.7	42.1	9.62	10.3	338	340	7.28	7.42	7.43	9.77	8.23	7.14	7.82	4.5	4.23	0.25	0.21	0.21
10	166	158	47.6	46.7	12.3	16.3	271	189	7.32	7.3	7.3	9.84	10.2	8.13	6.66	5.45	6.03	0.5	0.4	0.35
11	164	109	49	31.8	14.4	9.4	229	154	7.34	7.35	7.36	6.4	5.3	5	5.2	4.9	4.5	0.5	0.3	0.21
12	169	136	49	41.9	7.1	17	296	318	7.32	7.5	7.37	9	14	7.6	4.7	2.5	3.4	0.25	0.21	0.21
13	159	105	46	31.2	12.9	10.6	329	465	7.28	7.5	7.48	8.3	6.2	8	7.6	3	4.1	0.3	0.21	0.21
14	142	136	46	42	11.8	9.6	235	245	7.39	7.4	7.58	10.06	9.82	6.17	5.3	4.82	2.92	0.25	0.21	0.21
15	136	122	48	46.3	11.3	9.3	236	240	7.32	7.36	7.4	11.1	10.95	10.32	6.47	5.37	4.83	0.45	0.25	0.21
Mean	157	139	46.3	39.3	13.1	12.3	296.7	293.1	7.32	7.36	7.40	8.39	8.87	7.39	5.98	4.91	4.18	0.37	0.24	0.22
SD	22.04	25.5	6.18	9.30	6.4	3.03	56.70	97.88	0.06	0.08	0.07	2.31	2.86	1.54	1.28	1.5	0.82	0.10	0.05	0.034

**Table 2:** 

					APGAR		RDS grade	PS		Respiratory support				Complications						Duration of hospitalization (D)	Prognosis
	Sex	GA (weeks)	BW (g)	Delivery mode	1 minute	5 minute			Times	Duration of mechanical ventilation (D)	Duration of oxygen therapy (D)	Reintubation	Time return to BW (D)	BPD	VAP	IVH	ROP	LOS	NEC		
1	M	30 + 5	1630	CS	9	9	3	Y	1	5	5	N	20	Nil	Nil	Nil	Nil	Nil	Nil	18	Cured
2	M	30 + 5	1510	CS	5	9	4	Y	1	3	9	N	21	Nil	Nil	Nil	Nil	Nil	Nil	53	Cured
3	M	33	1630	VD	8	6	3	Y	1	4	11	N	36	Nil	Nil	Nil	Nil	Nil	Nil	46	Cured
4	M	31 + 2	1510	VD	9	10	2	Y	1	2.5	3	N	20	Nil	Y	Nil	Nil	Y	Nil	65	Cured
5	F	28 + 2	1400	CS	9	10	3	Y	1	4	9	Y	32	Nil	Y	Nil	Nil	Nil	Nil	30	Cured
6	M	30 + 1	1600	CS	8	10	4	Y	1	2.5	4	N	26	Nil	Y	Nil	Nil	Nil	Nil	4	Cured
7	M	29 + 1	1450	CS	9	10	2	Y	1	7	8	N	28	Nil	Nil	Nil	Nil	Nil	Nil	10	Cured
8	M	31 + 5	1700	CS	8	9	1	Y	1	2	2	N	14	Nil	Nil	Nil	Nil	Nil	Nil	47	Cured
9	M	32 + 5	1940	CS	9	10	2	Y	1	3	3	N	13	Nil	Nil	Nil	Nil	Nil	Nil	24	Cured
10	M	29 + 6	1400	CS	10	10	1	N	0	0	3	N	14	Nil	Y	Nil	Nil	Nil	Nil	82	Cured
11	M	32 + 5	1660	CS	9	10	3	Y	1	5.5	9	N	12	Nil	Nil	Nil	Nil	Nil	Nil	23	Cured
12	M	31	1240	VD	9	9	2	Y	1	4	5	N	14	Nil	Y	Y	Nil	Nil	Nil	20	Cured
13	M	30	1600	CS	9	10	3	Y	1	3	4.5	N	12	Nil	Nil	Nil	Nil	Nil	Nil	19	Cured
14	F	32 + 2	1450	CS	9	10	2	N	0	1.5	2.5	N	16	Nil	Nil	Nil	Nil	Nil	Nil	5	Cured
15	F	34 + 1	2220	VD	9	10	3	N	0	1	2	N	14	Nil	Nil	Nil	Nil	Nil	Nil	4	Cured
MEAN ± SD										3.2 ± 1.8	5.3 ± 3.0									30 ± 23.6	

**Table 3:** 

No	Volume collected	Volume postprocessing	Cells collected (108)	Cells postprocessing (108)	Cell concentration postprocessing∗106/ml	CFU-GM/105	CD34+/106	Viability	Infused NC∗107/kg	Infused age (H)	Infused volume	Pathogen detection
1	34	22	1.82	1.46	6.65	1.84	1.1	100	4.48	5	11	Negative
2	35	20	1.6	1.32	5.82	1.98	0.7	99.5	5	6	13	Negative
3	61	24	2.44	1.67	6.95	2.3	1.14	99.8	5	7	12	Negative
4	33	16	0.97	0.86	5.4	0.72	0.31	99.8	5	4	14	Negative
5	36	22	1.43	1.21	5.5	1.57	0.59	99.8	5	9	13	Negative
6	28	16	1.43	1.23	7.7	1.53	0.9	99.5	5	4.5	11	Negative
7	55	28	5.81	5.43	26.3	11.27	5.69	99.8	5	6	3	Negative
8	65	20	2.99	2.37	11.85	1.66	1.66	99.9	5	7	8	Negative
9	81	20	6.14	4.33	21.65	9.96	3.9	99.9	5	8	5	Negative
10	27	28	2.21	1.97	7.05	3.35	0.55	99.8	5	8	28	Negative
11	46	24	2.96	2.65	13.6	3.32	1.01	99.5	5	7	6	Negative
12	37	24	1.51	1.4	5.85	1.04	0.08	99.9	5	9	12	Negative
13	24	22	1.72	1.58	8.53	2.82	1.1	99.8	5	6	9	Negative
14	69	24	8.11	7.83	40.8	6.08	16.22	99.8	5	7	2	Negative
15	76	30	5.3	4.7	22.8	6.39	1	99.5	5	8	7	Negative
Mean	47.13	22.67	3.10	2.67	13.10	3.72	2.40	99.7	4.97	6.77	10.27	
SD	19.10	4.05	2.17	2.00	10.35	3.25	4.10	0.17	0.13	1.52	6.18	

## Data Availability

The data used to support the findings of this study are available from the corresponding author upon request.
